# MicroRNA-Mediated Direct Reprogramming of Human Adult Fibroblasts Toward Cardiac Phenotype

**DOI:** 10.3389/fbioe.2020.00529

**Published:** 2020-06-05

**Authors:** C. Paoletti, C. Divieto, G. Tarricone, F. Di Meglio, D. Nurzynska, V. Chiono

**Affiliations:** ^1^Department of Mechanical and Aerospace Engineering, Politecnico di Torino, Turin, Italy; ^2^Istituto Nazionale di Ricerca Metrologica, Advanced Materials Metrology and Life Science, Turin, Italy; ^3^Department of Public Health, University of Naples Federico II, Naples, Italy

**Keywords:** cardiac fibroblasts, direct reprogramming, cardiomyocytes, microRNAs, digital droplet PCR (ddPCR)

## Abstract

Modulation of microRNA expression holds the promise to achieve direct reprogramming of fibroblasts into cardiomyocyte-like cells as a new strategy for myocardial regeneration after ischemic heart disease. Previous reports have shown that murine fibroblasts can be directly reprogrammed into induced cardiomyocytes (iCMs) by transient transfection with four microRNA mimics (miR-1, 133, 208, and 499, termed “miRcombo”). Hence, study on the effect of miRcombo transfection on adult human cardiac fibroblasts (AHCFs) deserves attention in the perspective of a future clinical translation of the approach. In this brief report, we studied for the first time whether miRcombo transient transfection of AHCFs by non-viral vectors might trigger direct reprogramming of AHCFs into cardiomyocyte-like cells. Initially, efficient miRNA delivery to cells was demonstrated through the use of a commercially available transfection agent (DharmaFECT1). Transient transfection of AHCFs with miRcombo was found to upregulate early cardiac transcription factors after 7 days post-transfection and cardiomyocyte specific marker cTnT after 15 days post-transfection, and to downregulate the expression of fibroblast markers at 15 days post-transfection. The percentage of cTnT-positive cells after 15 days from miRcombo transfection was ∼11%, as evaluated by flow cytometry. Furthermore, a relevant percentage of miRcombo-transfected AHCFs (∼38%) displayed spontaneous calcium transients at 30 days post-transfection. Results evidenced the role of miRcombo transfection on triggering the trans differentiation of AHCFs into iCMs. Although further investigations are needed to achieve iCM maturation, early findings from this study pave the way toward new advanced therapies for human cardiac regeneration.

## Introduction

Myocardial infarction (MI) remains one of the leading causes of death globally. After MI, the injured area of the heart undergoes a deep tissue remodeling: cardiomyocytes (CMs) death, extracellular matrix (ECM) degradation and increased myocardial fibrosis dramatically impair cardiac function, leading to heart failure (van den Borne et al., 2010). The only available therapeutic strategy against heart failure is heart transplantation. Hence, cardiac regeneration is still a major clinical challenge.

An ideal cardiac regenerative medicine strategy should replace lost CMs and recover myocardial contractility. Recently, efforts have been addressed at reducing the extent of cardiac fibrotic scar, as it reduces the contractile heart functionality (Talman and Ruskoaho, 2016). Direct reprogramming of fibroblasts into induced cardiomyocytes (iCMs) represents a new emerging strategy to convert cardiac fibrotic tissue into functional tissue (Bektik and Fu, 2019). Ieda et al. (2010) were the first to report direct reprogramming of mouse fibroblasts into beating iCMs through a simultaneous overexpression of GATA binding protein 4 (*GATA4*), Myocyte enhancer factor 2C (*MEF2C*), T-box 5 (*TBX5*) cardiac transcription factors (TFs) both *in vitro* and *in vivo*. Later, Song et al. (2012) added Heart and Neural Crest derivatives expressed 2 (*HAND2*) to the previous combination of cardiac TFs, increasing reprogramming efficiency of mouse fibroblasts *in vitro* and improving cardiac function of injured hearts *in vivo*. Since then, several other direct reprogramming strategies have been reported for mouse and human fibroblasts, generally based on gene therapy via viral overexpression of specific cardiac TFs (Chen et al., 2017).

Efforts are now addressed to develop a direct reprogramming method which is safe and free from ethical and technical concerns associated with genetic manipulation of cells. The use of microRNAs (miRNAs) may represent one valid strategy to induce direct reprogramming of human fibroblasts into iCMs, satisfying such requirements. miRNAs are short non-coding RNAs (of approximately 22 nucleotides) that regulate gene expression post-transcriptionally (He and Hannon, 2004). As cardiac tissue-specific miRNAs play pivotal role in cardiac development and functions, the regulation of their expression would aid in generating functional iCMs starting from human fibroblasts (Tian et al., 2017). Endogenous miRNAs can be easily overexpressed or downregulated through the administration of miRNA mimics or miRNA inhibitors, respectively (Peng et al., 2015). MiRNA mimics and inhibitors hold a great potential as therapeutic agents because of their cytoplasmic activity, relatively small size and ability to be administered systemically or locally by nanoparticle-based delivery systems, avoiding the use of viral vectors (Lee et al., 2019). Modulation of miRNAs for direct reprogramming was first proposed by Jayawardena et al. (2012), who demonstrated that a combination of four miRNA mimics (referred to as “miRcombo”: miR-1, 133, 208 and 499) promotes the direct reprogramming of mouse fibroblasts into iCMs both *in vitro* and *in vivo*. The selected miRNAs are highly conserved in cardiac and skeletal tissue and have been found to regulate developmental and physiological processes in the heart (van Rooij et al., 2009; Xu et al., 2011). Furthermore, this specific set of microRNAs was found to be expressed in human embryonic stem cells-derived CMs, whereas their expression was nearly undetectable in fibroblasts (Kitchener et al., 2010).

Although the potentiality of miRcombo in reprogramming mouse fibroblasts into iCMs has been demonstrated (Jayawardena et al., 2012, 2015), the translation of miRcombo-mediated direct reprogramming from mouse to human cells is not straightforward and deserves further investigation. Indeed, human adult fibroblasts are intrinsically more resistant to direct reprogramming than mouse fibroblasts, as demonstrated by those direct reprogramming strategies, first developed on mouse fibroblasts, which failed on human fibroblasts or required substantial adjustments (Ieda et al., 2010; Nam et al., 2013).

In this brief research report, we sought to determine if miRcombo transient transfection of AHCFs was able to initiate the trans-differentiation process of AHCFs toward cardiomyocyte-like cells. After initial assessment of efficient miRNA delivery to AHCFs, cells were transfected with miRcombo, showing increased expression of cardiomyocyte markers and down-regulated expression of fibroblast markers after 7–15 days post-transfection. Moreover, miRcombo treated cells exhibited spontaneous calcium oscillation after 30 days from transfection.

Early findings presented in this brief research report pave the way toward future new advanced approaches for human cardiac regeneration, through *in situ* direct reprogramming of cardiac fibroblasts populating post-infarct scar into cardiomyocyte-like cells.

## Materials and Methods

### Cell Culture

Normal human atrial cardiac fibroblasts (AHCFs) were purchased from Lonza (CC-2903; batch number: 0000662121; donor characteristics: male; 48 years old; passage number: 2) and maintained in culture using Fibroblasts Growth Medium-3 (Lonza, CC-4526) containing 10% fetal bovine serum (FBS), 1% insulin, 1% human basal fibroblast growth factor (hFGF-B) and 1% gentamicin. Cells were expanded until passage four and then used for experiments.

### MiRNA Transient Transfection

AHCFs were plated either in six-well plates at 10 × 10^5^ cells/well (for RNA isolation and flow cytometry experiments), or in 35 mm μ-dish (Ibidi) at 9 × 10^5^ cells/well (for calcium transient analysis) in Dulbecco’s Modified Eagle Medium (DMEM) High Glucose (Gibco) with 10% FBS (Sigma-Aldrich) and 1% glutamine (Sigma-Aldrich) 1 day before transfection.

To initially study miRNA internalization and mRNA target downregulation by AHCFs, cells were transfected using DharmaFECT1 (Dharmacon) with miR-1 (miR-1-3p, mirVana miRNA mimic, Life Technologies) or negmiR (Negative Control #1, mirVana miRNA Mimic, Life Technologies), at a final concentration of 25 nmol/L according to manufacturer’s instructions, in DMEM High Glucose with 10% FBS and 1% L-glutamine. After 24 h, the medium was replaced with DMEM High Glucose with 15% FBS, 1% glutamine and 1% penicillin/streptomyocin/ampicillin (Lonza). Culture was continued up to 48 h.

Then, AHCFs were transfected using DharmaFECT1 with miRcombo (miR-1-3p, miR-133a-3p, miR-208a-3p, and miR-499a-5p, mirVana miRNA mimic, Life Technologies) or negmiR (Negative Control #1, mirVana miRNA Mimic, Life Technologies), at a final concentration of 25 nmol/L according to manufacturer’s instructions, in DMEM High Glucose (Gibco) with 10% FBS and 1% glutamine. After 24 h, medium was replaced with DMEM High Glucose with 10% FBS, 1% glutamine and 1% penicillin/streptomyocin/ampicillin (Lonza). Culture was continued up to 30 days.

### RNA Isolation and Droplet Digital PCR

Total RNA was extracted using QIAzol Lysis Reagent (Qiagen) according to manufacturer’s instructions. RNA concentration and quality were assessed using NanoQuant plate (Tecan Group Ltd). cDNA (200 ng) was obtained using High Capacity cDNA Reverse Transcription Kit (Applied Biosystems).

To study miR-1 uptake by cells, miRNA reverse-transcription PCR was performed using miRCURY LNA RT Kit (Qiagen) at 24 h (i.e., immediately after transfection) and 48 h (i.e., at 24 h from transfection) culture times. RNA samples were diluted to 5 ng/μl in nuclease-free water and the reaction was prepared following the manufacturer’s instructions. Droplet digital PCR (ddPCR, Bio-Rad Laboratories) was performed to assess the expression of miR-1 (Hsa-miR-1-3p miRCURY LNA miRNA PCR Assay) using EvaGreen supermix (Bio-Rad Laboratories). Droplet generation was performed according to manufacturer’s instructions. Thermal-cycling conditions were: 95°C for 5 min (1 cycle), 95°C for 30 s and 55°C for 1 min (40 cycles), 90°C for 5 min (1 cycle), and a 4°C infinite hold. PCR plate was loaded on Bio-Rad QX100 droplet reader for quantification of cDNA copies/μL. Analysis of the ddPCR data was performed by QuantaSoft analysis software (Bio-Rad Laboratories). No template control with water was included in each assay. Experiments were performed in triplicate and repeated three times.

Downregulation of *TWF-1* target after miR-1 transfection was analyzed at 24 h (i.e., immediately after transfection) and 48 h (i.e., at 24 h from transfection) culture times. The expression of *TWF-1* (ID assay: dHsaCPE5028122) was analyzed by ddPCR, using ddPCR supermix for probes without dUTP.

Similarly, after miRcombo or negmiR transfection, the expression of *GATA4* (ID assay: dHsaCPE5050488), *MEF2C* (ID assay: dHsaCPE5050696), *TBX5* (ID assay: dHsaCPE5048560), *HAND2* (ID assay: dHsaCPE5049426) and *NKX2.5* (ID assay: dHsaCPE5042098) was evaluated at 7 days post-transfection, while the expression of *TNNT2* (ID assay: dHsaCPE5052344), *TNNI3* (ID assay: dHsaCPE5050934), *VIM* (ID assay: dHsaCPE5032314), *DDR2* (ID assay: dHsaCPE5048156) and *FSP-1* (ID assay: dHsaCPE5042942) was assessed at 15 days post-transfection, using ddPCR supermix for probes without dUTP. Glyceraldehyde 3-phosphate dehydrogenase (*GAPDH*; ID assay: dHsaCPE5031597) was used as a housekeeping gene to perform quantitative normalization.

For *TWF-1* and cardiac markers analysis, droplet generation was performed according to manufacturer’s instructions. Thermal-cycling conditions were 95°C for 10 min (1 cycle), 94°C for 30 s and 55°C for 30 s (40 cycles), 98°C for 10 min (1 cycle), and a 4°C infinite hold.

In all ddPCR experiments, PCR plate was loaded on Bio-Rad QX100 droplet reader for quantification of cDNA copies/μL. Analysis of the ddPCR data was performed by QuantaSoft analysis software (Bio-Rad Laboratories). No template control with H_2_O was included in each assay. Experiments were performed in triplicate and repeated three times.

Results are reported as the concentration (cDNA copies/μL) of the gene of interest calculated on the concentration mean (cDNA copies/μL) of *GAPDH*.

### Flow Cytometry

After transfection with miRcombo or negmiR, AHCFs were cultured for additional 15 days. Then, cells were trypsinized with 0.05% Trypsin/EDTA (Sigma Aldrich) and permeabilized with 0.5% v/v Tween 20 in phosphate buffered saline (PBS) for 5 min. Ice cold PBS with 10% FBS and 1% sodium azide (Sigma Aldrich) was used for washing between each step. Cells were incubated with Cardiac Troponin T primary antibody (cat #701620, Invitrogen) for 1 h at 4°C and Alexa Fluor 488-conjugated secondary antibody (ab150077, Abcam) for 1 h at 4°C in the dark. Cells were run on Guava EasyCyte (Merck) flow cytometer and data analysis was performed using GuavaSoft 3.2.

### Measurements of Spontaneous Calcium Transients

Calcium transient was analyzed in AHCFs after 30 days post-transfection with miRcombo or negmiR. To record calcium transients, cells were loaded with 5μM of Fluo-4 AM (Invitrogen) in modified Tyrode’s solution (140 mM NaCl, 5 mM KCl, 1.8 mM CaCl_2_, 1 mM MgCl_2_, 10 mM glucose and 10 mM Hepes) with 0.1% bovine serum albumin (BSA) at 37°C for 20 min while shielded from light. Cells were washed in modified Tyrode’s solution at 37°C for 10 min. Calcium transient was recorded using Nikon Eclipse Ti2 spinning disk and NIS-Elements software (Nikon). At least 4 fields were observed for each sample. Data were analyzed using ImageJ software (NIH) and Ca^2+^ level was reported as F/F_0_ ratio, where F is the intensity of fluorescence emission recorded for each cell, while F_0_ is the background fluorescence.

### Statistical Analysis

The results are shown as means ± standard error of the mean (SEM) of replicate experiments. Statistical analyses were performed with Student’s *t*-test, with p-value reported as ^∗^*p* < 0.05 considered statistically significant, ^∗∗^*p* < 0.01 considered highly significant and ^∗∗∗^*p* < 0.0001 very highly significant. All graphs were prepared using GraphPad software.

## Results

**FIGURE 1:**
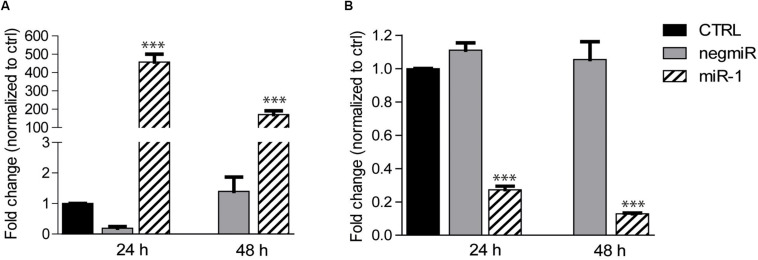
DharmaFECT1 mediated delivery of miR-1 and *TWF-1* target downregulation in AHCFs. **(A)** Fold change of miR-1 expression (relative to untransfected control) in AHCFs transfected with negmiR or miR-1 using DharmaFECT1 at 24 h (i.e., immediately post-transfection) and 48 h (i.e., 24 h post-transfection) culture times, analyzed by ddPCR. Data are representative of three independent experiments. Stated *p*-value ^∗∗∗^*p* < 0,001 is reported versus negmiR control. **(B)** Fold change of *TWF-1* mRNA target expression (relative to untransfected control) in AHCFs transfected with negmiR or miR-1 using DharmaFECT1 at 24 h (i.e., immediately post-transfection) and 48 h (i.e., 24 h post-transfection) culture times. Data are representative of three independent experiments. Stated *p*-value ^∗∗∗^*p* < 0,001 is reported versus negmiR control. CTRL, untransfected control.

We initially studied the efficiency of DharmaFECT1 in delivering miRNAs to AHCFs, using miR-1 as a model miRNA, as compared to a non-targeting miRNA negative control (negmiR). DdPCR analysis of miR-1 transfected cells showed relative overexpression of miR-1, and knockdown of *TWF-1* target gene in AHCFs, at 24 and 48 h culture times (Figures 1A,B).

**FIGURE 2:**
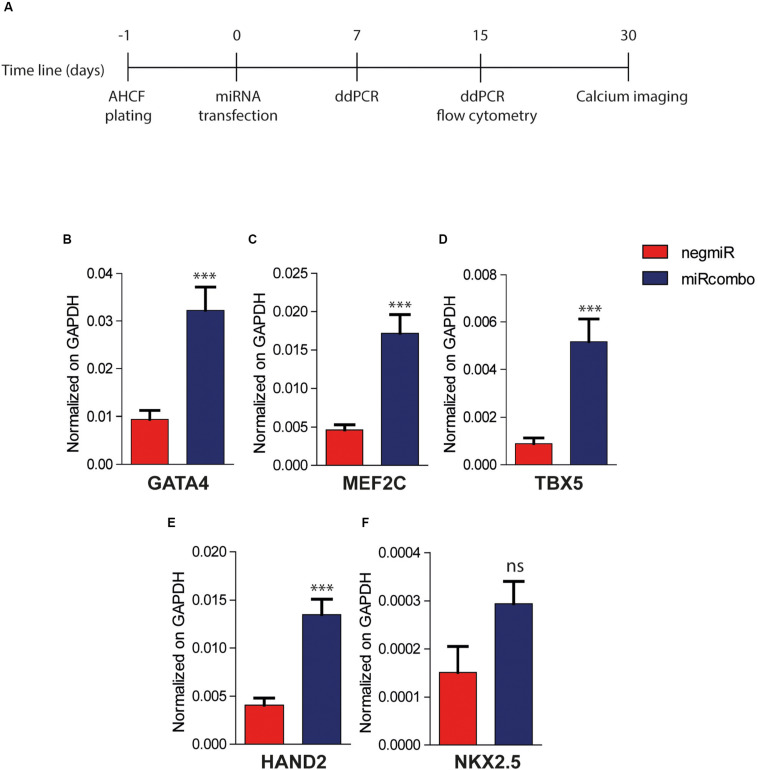
MiRcombo-transfected AHCFs show increased cardiac transcription factor expression. **(A)** Representative scheme of experimental design. AHCFs were transfected with miRcombo or negative control (negmiR). The acquisition of cardiomyocyte-associated features was evaluated after 7 days (cardiac transcription factors), 15 days (cardiomyocyte and fibroblast markers) and 30 days (calcium transients). **(B–F)** Gene expression of cardiac transcription factors. The expression of *GATA4*, *MEF2C*, *TBX5*, *HAND2*, and *NKX2.5* was evaluated by ddPCR 7 days post transfection in AHCFs transfected with miRcombo (red) or negmiR (blue). Data are representative of three independent experiments, each performed in triplicate. Stated *P*-value is versus negmiR controls.

Then, the effect of miRcombo transient transfection on AHCFs trans-differentiation into iCMs was evaluated at different time points by different analyses depicted in Figure 2A. As a negative control, AHCFs were also transfected with negmiR.

DdPCR analysis showed that miRcombo transfected cells upregulated the expression of early cardiac transcription factors (TFs), with a significantly increased expression of *GATA4* (*p* = 0.007), *MEF2C* (*p* = 0.0004), *TBX5* (*p* < 0.0001) and *HAND2* (*p* = 0.0001) cardiac TFs, compared to negmiR transfected cells, 7 days after transfection (Figures 2B–E). Additionally, the expression of *NKX2.5* cardiac TF was increased in miRcombo transfected cells compared to controls, although not significantly (Figure 2F).

**FIGURE 3:**
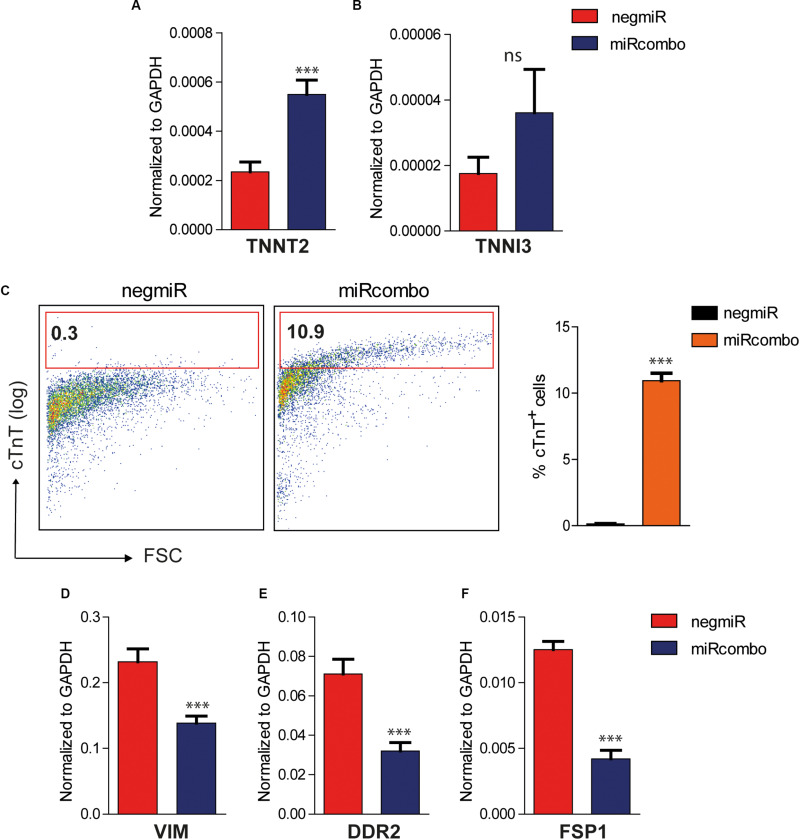
Induction of cardiomyocyte markers in miRcombo-transfected AHCFs. **(A,B)** Gene expression of cardiomyocyte markers *TNNT2* and *TNNI3* in AHCFs transfected with miRcombo (red) or negmiR (blue) evaluated by ddPCR. Data are representative of three independent experiments, each performed in triplicate. **(C)** Representative flow plots (left panel) and percentage (right panel) of cTnT positive cells in AHCFs transfected with miRcombo (*n* = 3) and negmiR (*n* = 3) 15 days after transfection. **(D–F)** Gene expression of fibroblastic markers *VIM*, *DDR2*, and *FSP-1* in AHCFs transfected with miRcombo (red) or negmiR (blue) by ddPCR. Data are representative of three independent experiments, each performed in triplicate. Stated *P*-value is versus negmiR.

As a further step, the expression of mature cardiomyocyte markers was evaluated in miRcombo transfected AHCFs at 15 days after transfection. The expression of *TNNT2*, which encodes for cardiac troponin T (cTnT), was significantly increased in miRcombo-transfected cells compared to negmiR controls (*p* = 0.0004) (Figure 3A). Also, *TNNI3* expression was slightly increased in miRcombo transfected cells compared to controls, although non-significantly (Figure 3B).

The reprogramming efficiency of miRcombo-transfected cells was evaluated using flow cytometry. Notably, approximately 11% of fibroblasts were cTnT^+^ after 15 days culture time from miRcombo transfection, whereas very low percentage of cTnT^+^ cells (0.3%) was measured in negmiR transfected cells (Figure 3C).

Along with the upregulation of cardiomyocyte markers, ddPCR analysis showed that the expression of fibroblast markers, such as vimentin (*VIM*, *p* = 0,0003), discoidin domain receptor-2 (*DDR2*, *p* = 0,0004) and fibroblast specific protein-1 (*FSP-1*/*S100A4*, *p* < 0,0001) was significantly downregulated in miRcombo-transfected cells compared to controls after 15 days culture time from miRcombo transfection (Figures 3D–F).

Overall, the results suggested that, after 15 days from reprogramming induction, miRcombo-transfected cells started to display functional cardiomyocyte-specific markers along with a reduced fibroblast phenotype.

**FIGURE 4:**
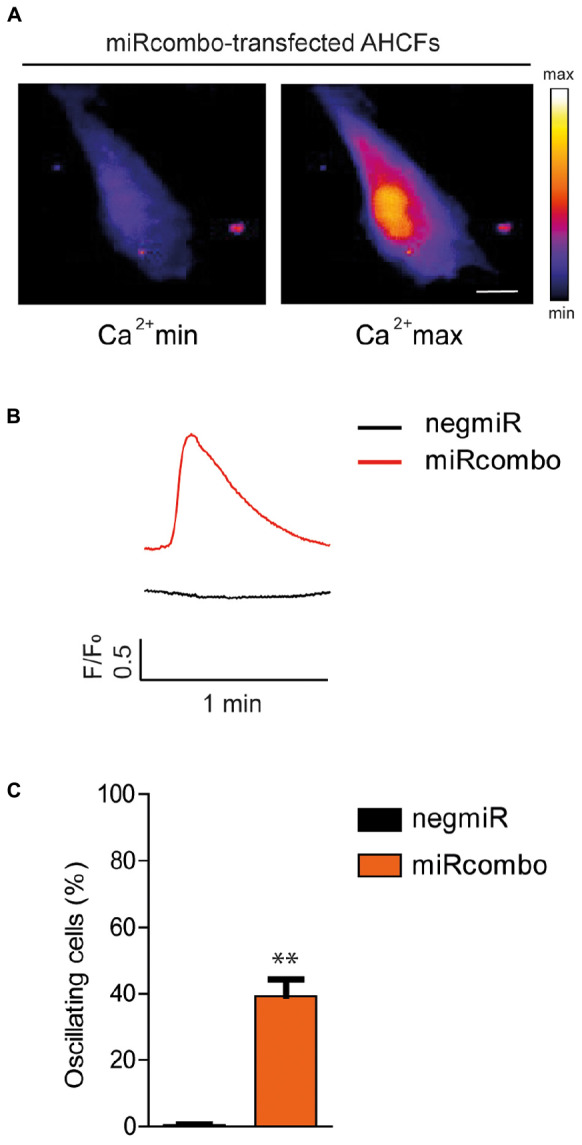
miRcombo-transfected cells exhibit calcium transients. **(A,B)** Representative image, recorded with fluorescence microscopy **(A)** and trace **(B)** of Ca^+2^ oscillations in AHCFs transfected with miRcombo (*n* = 3) and negmiR (*n* = 3) after 30 days from transfection. Scale bar: 25 μm. **(C)** Bar graph reports the percentage of cells showing Ca^+2^ transients after 30 days from miRcombo and negmiR transfection. Stated *P*-value is versus negmiR.

Finally, to determine whether miRcombo-transfected AHCFs exhibited functional cardiomyocyte properties after a long period of culture, we examined intracellular calcium oscillation at 30 days after transfection. Results showed that miRcombo transfection triggered calcium oscillation, lasting nearly 1 min, in ∼38% of total cells, while rare or no calcium oscillation was found in negmiR transfected control cells (Figures 4A–C).

In conclusion, results demonstrated that miRcombo-transfection triggered transdifferentiation of AHCFs into cardiomyocyte-like cells, also increasing functional cardiomyocyte properties compared to AHCFs transfected with negmiR.

## Discussion

Direct reprogramming of human cardiac fibroblasts into cardiomyocytes represents an attractive approach to revert cardiac fibrosis and recover heart function after MI (Chen et al., 2017), avoiding the need for cell-based therapies to replace cardiomyocytes (Hashimoto et al., 2018). Cardiac fibrotic scar is mainly composed by activated fibroblasts (myofibroblasts), while the target healthy cardiac tissue contains 20% cardiac fibroblasts and 35% cardiomyocytes (Gray et al., 2018). Ideally, through direct reprogramming, a suitable number of fibroblasts in the scar would be converted into cardiomyocytes to re-establish cell-cell connections and functionality of healthy cardiac tissue.

Since the first evidence of direct reprogramming of fibroblasts into cardiomyocytes in 2010 (Ieda et al., 2010), several efforts have been performed to directly reprogram fibroblasts into iCMs, using combinations of cardiac transcription factors (Song et al., 2012), microRNA mimics (Jayawardena et al., 2012), and small molecules (Fu et al., 2015). However, most direct reprogramming methods have been generally optimized on mouse cells, which does not assure their efficacy on AHCFs (Chen et al., 2017). Indeed, AHCFs are known to be more resistant to cellular reprogramming than murine fibroblasts due to epigenetic barriers that hinder phenotypic changes (Talkhabi et al., 2017).

Successful direct reprogramming of AHCFs into iCMs was achieved by inducing the expression of cardiac TFs and miRNAs, through retroviruses or inducible lentiviruses (Nam et al., 2013; Muraoka et al., 2014), which cause stable and heritable integration of a specific nucleic acid sequence into the target cell genome (Chen et al., 2017). As an example, Nam et al. (2013) directly reprogrammed AHCFs into iCMs with an efficiency of ∼13% (measured by cTnT^+^ cells at 2 weeks) by retroviral overexpression of miR-1, miR-133 and specific cardiac TFs. On the other hand, Muraoka et al. (2014) found out ∼20% of cTnT^+^ cells after 1 week from simultaneous transfection of AHCFs with different TFs (by lentiviral vectors) and miR-133 (by Lipofectamine 2000), however, they used relatively young AHCFs (average age: 35 years old) and did not analyse cell functional properties. Overall, although effective, viral vectors suffer from potential risks which currently hinder an *in situ* clinical application (Labatut and Mattheolabakis, 2018).

MiRcombo, a combination of four miRNA mimics (miRs-1, 133, 208, and 499) has been previously demonstrated to successfully reprogram mouse neonatal fibroblasts into functional cardiomyocytes *in vitro*, and to regenerate cardiomyocytes *in vivo* after myocardial injury (Jayawardena et al., 2012, 2015). On the other hand, the effect of miRcombo transfection on human fibroblasts has never been tested. In the perspective of a future clinical application of miRcombo approach, preclinical studies should be carried out using human cells.

In this work, DharmaFECT1 was demonstrated to be an efficient agent for miRNA delivery to AHCFs (Figures 1A,B). Moreover, miR-1 delivery to AHCFs efficiently induced knockdown expression of *TWF-1* (miR-1 target in cardiac fibroblasts), which was maintained up to 48 h culture time.

Then, DharmaFECT1 was employed for transfection of AHCFs with miRcombo or negmiR, and cardiac gene and protein expression of transfected AHCFs was investigated at different time steps. After 7 days from transient transfection with miRcombo, AHCFs showed upregulation of early cardiac TF expression, such as *GATA4*, *MEF2C*, *TBX5*, *HAND2*, respect to negmiR transfected cells (Figure 2). Early cardiac TFs are known to work in synergy, guiding cardiac cell fate and contractile protein expression (Olson, 2006). Nuclear localization of cardiac TFs was found in chemically induced cardiomyocytes from mouse fibroblasts (Fu et al., 2015). In the future, immunostaining of cardiac TFs in miRcombo-transfected cells could help in understanding their correct location in cell nuclei, ensuring their function in inducing mature cardiomyocyte marker expression.

Then, the expression of functional and structural cardiomyocyte markers in AHCFs at longer time from transfection was evaluated. Particularly, gene expression of *TNNT2* was enhanced in miRcombo transfected AHCFs respect to negmiR transfected cells after 15 days from transfection (Figure 3A). These results were also confirmed by flow cytometry, showing a reprogramming efficiency – measured as percentage of cTnT^+^ cells - of ∼11% (Figure 3C), which was indeed higher than the value reported for miRcombo-transfected neonatal murine cardiac fibroblasts (∼3%) (Li et al., 2016). On the other hand, the measured reprogramming efficiency was similar to values reported in previous investigations on direct reprogramming of AHCFs into iCMs (∼13% after 2 weeks reported by Nam et al., 2013).

Additionally, a reduced expression of fibroblastic markers (*VIM*, *DDR2* and *FSP-1*) was measured after 15 days of culture post-transfection (Figures 3D–F).

Notably, differently from previous studies on miRcombo transfection of mouse neonatal fibroblasts (Jayawardena et al., 2012; Dal-Pra et al., 2019), miRcombo-transfected AHCFs did not show sarcomeric organization after 15 days from miRcombo transfection, suggesting an immature phenotype (data not shown). On the other hand, previous studies reported early sarcomeric structure in AHCFs transfected with cardiac TFs and miRNAs, after at least 4–5 weeks from transfection (Nam et al., 2013; Muraoka et al., 2014). Compared to neonatal fibroblasts, AHCFs are more resistant to direct reprogramming, caused by their stable epigenetic program. For this reason, in the future, longer culture times and/or additional stimulating factors will be applied to transdifferentiate AHCFs into mature iCMs.

Upon maintaining miRcombo-transfected AHCFs in culture for 30 days, ∼38% of total cells exhibited spontaneous calcium oscillation, suggesting cardiomyocyte-like functionality (Figures 4A–C). This is a relevant result when compared to what reported by Nam et al. (2013) who found out a cell percentage of ∼15% exhibiting calcium transients induced by KCl stimulation, after 4 weeks from transfection. Although most of the previous studies on direct cardiac reprogramming analyzed calcium transient through stimulation with high KCl concentration or caffeine (Fu et al., 2013; Nam et al., 2013), a few authors reported the analysis of spontaneous calcium oscillation of iCMs as in our case (Smith et al., 2013). In the future, induced calcium oscillation through KCl or caffeine stimulation will be analyzed to confirm cardiomyocyte-like functionality.

In conclusion, results demonstrated for the first time that miRcombo transfection was able to activate a phenotypic switch of AHCFs toward cardiomyocyte-like cells. Although other methods are under study to directly reprogram AHCFs into iCMs (Nam et al., 2013; Muraoka et al., 2014), the advantage of miRcombo-mediated approach derives from the possibility for non-viral transfection, overcoming the hurdles associated with viral vectors, such as high cost, difficulties in large scale production and potential mutagenesis and immunogenic behavior (Labatut and Mattheolabakis, 2018). Indeed, safety represents one key requirement for perspective application of direct reprogramming to humans. In the present study, the overexpression of defined cardiac developmental factors and mature markers was achieved by transient transfection with miRcombo through a commercially available lipidic transfection agent. AHCFs transfected with miRcombo were able to maintain the expression of specific cardiomyocyte markers at the tested time points (7 and 15 days). Future studies will be addressed to thoroughly study miRcombo-mediated direct reprogramming of AHCFs at longer times and under different microenvironmental stimulations, and to develop non-viral vectors for *in situ* transfection with miRcombo, paving the way toward a clinical translation of direct reprogramming strategies (Muraoka et al., 2014; Li et al., 2016; Paoletti et al., 2018).

## Conclusion

This brief report provides a preliminary study showing that a combination of four microRNAs (miRcombo) is able to drive a significant change in human fibroblast phenotype, starting their trans-differentiation toward cardiomyocyte-like cells. Despite further investigations are required to confirm and to enhance the functional activity of iCMs, the present work demonstrates that miRcombo transfection is effective in directly reprogramming AHCFs into cardiomyocyte-like cells with potentialities for future clinical application.

## Data Availability Statement

The datasets generated for this study are available on request to the corresponding author.

## Author Contributions

CP designed the experimental part of the project, conducted experiments, and analyzed the data. CD supported CP in the *in vitro* culture experiments and trained CP in ddPCR analysis at INRIM biology lab. GT supported CP in ddPCR analysis for miR-1 overexpression and mRNA target downregulation. FD and DN supported CP in the *in vitro* culture experiments with adult human fibroblasts. VC supervised the work and acquired funding. The manuscript was written through main contributions by CP and VC and further help by all the authors. All authors have given approval to the final version of the manuscript.

## Conflict of Interest

The authors declare that the research was conducted in the absence of any commercial or financial relationships that could be construed as a potential conflict of interest.
